# Health sciences librarian competency perceptions: a survey of national community college librarians

**DOI:** 10.5195/jmla.2021.994

**Published:** 2021-01-01

**Authors:** Sandra C. McCarthy

**Affiliations:** 1 mccarthy@wccnet.edu, Faculty Librarian, Bailey Library, Washtenaw Community College, Ann Arbor, MI

## Abstract

**Objective:**

The primary objective of this study was to determine how community college health sciences librarians perceive their proficiencies in the essential skills, knowledge, and abilities necessary for the practice of a health information professional as defined by the Medical Library Association (MLA) *Competencies for Lifelong Learning and Professional Success.* A secondary objective was to determine their current level of engagement with the professional community and identify barriers to further professional development.

**Methods:**

A survey was posted to various email discussion lists, and volunteer follow-up interviews were conducted.

**Results:**

The survey was completed by seventy-five community college health sciences librarians, and seven follow-up interviews were performed. Survey results indicated that community college health sciences librarians perceived themselves as having intermediate or advanced intermediate proficiency in the six MLA competencies. Survey and interview results indicated that community college health sciences librarians were engaged with the profession and faced the same barriers to continued professional development and continued education as other academic librarians.

**Conclusion:**

The results affirm that community college librarians who are responsible for collections and services in the health sciences meet the MLA competencies, which fills a gap in the literature regarding how these librarians develop professional competencies and are involved in professional associations. The results suggest that community college librarians can improve their skill levels by continuing their education and following trends in the literature.

## INTRODUCTION

Community colleges exert a tremendous influence in the higher education environment of the United States. In the 2018/19 academic year, US community colleges awarded 852,504 associate degrees and 579,822, certificates [1]. Of the 12 million students enrolled at a community college in fall 2018, 6.8 million (53%) enrolled in credit courses and 5 million (47%) enrolled in noncredit offerings [1], with the American Association of Community Colleges reporting that approximately 136,300 certificates and 124,700 associate degrees were awarded in health sciences disciplines [2]. Thus, librarians in community colleges play an integral role in designing, curating, and delivering health sciences collections and services to their students and faculty. In addition, community college librarians and libraries constitute an essential component of the accreditation review processes for nursing and health sciences programs.

To adequately support student learners, faculty, and health sciences programs, it is vital for librarians who are responsible for health sciences collections and services to demonstrate professional competency. The term “competency” was introduced in the 1970s to measure job success [3] and can be assessed by knowledge, skills, abilities, traits, and behaviors [4]. In 2017, the Medical Library Association (MLA) revised its *Competencies for Lifelong Learning and Professional Success,* which had originally been developed in 2007, to reflect current challenges facing librarians and librarianship. These competencies “identify essential professional skills and abilities that can be observed, measured, and taught” for all health sciences librarians regardless of work setting [5]. Therefore, the MLA competencies can guide community college librarians toward determining and improving their knowledge, skills, and abilities in delivering services to user populations in the health sciences.

Previous studies of competencies or standards in health sciences librarianship examined early career professionals, academic librarians at four-year institutions, and hospital librarians. These studies reported that newly hired health sciences librarians could develop their skills and competencies by learning on the job [6] and that librarians who continued their education would keep abreast of new trends and technology and ultimately stay current and competitive in the profession [7]. Early career health sciences librarians who self-reported attainment of the 2007 MLA competencies felt most confident with “Health Sciences Information Services” and least confident in “Research, Analysis, and Interpretation” [8]. Therefore, a rigorous study of MLA competencies applied to the community college context is needed because community college health sciences librarians are often hired without existing subject expertise and are expected to develop skills on the job.

A considerable volume of literature speaks to barriers that librarians face in meeting competencies set by professional organizations through continuing education opportunities. Identified barriers include the cost of attending face-to-face professional programs, travel distance, lack of time and staffing, and budget cuts [9, 10]. Also, health sciences librarians engaging in research face barriers such as lack of training and research skills, lack of institutional support, and personal time commitment [11].

This study addresses three research questions:

- How do community college health sciences librarians perceive their competencies in professional skills and abilities?
- What barriers do community college health sciences librarians face in developing their competencies?
- How engaged are community college health sciences librarians in attending conferences, continuing education, and presenting or publishing?

## METHODS

### Instrument

This study received approval from the Washtenaw Community College Institutional Review Board (IRB). The author, a member of the MLA Research Training Institute (RTI) 2019 cohort, designed a forty-nine-question survey that included questions about demographics, competencies, obstacles, and engagement with the profession (supplemental Appendix A). The competencies section utilized a Likert scale for each of the MLA competencies and sub-competencies with 1 indicating novice or no knowledge, 2 indicating low or beginner proficiency, 3 indicating intermediate proficiency, 4 indicating advanced intermediate proficiency, and 5 indicating expertise. Not all survey questions were required, and respondents were allowed to skip questions. The survey was pretested by eleven health sciences librarians from universities and community colleges to review the questions and length of time it would take to complete the survey. After incorporating their feedback, the revised survey was deployed using Springshare LibWizard in January 2020.

### Procedures

The email discussion lists that were selected for respondent recruitment included state and national health association lists as well as a national community college email discussion list (CJCLS-L). Health sciences libraries email discussion lists included the MLA Nursing and Allied Health Resources and Services (NAHRS-L) Caucus list, all regional MLA chapter lists [12], and state library health sciences association lists. The survey was also posted to select American Library Association (ALA) community discussion boards via ALA Connect and to the Tribal College Librarians Institute list (TCLI-L). This wide distribution was needed given the dispersed nature of community college librarians in large and small institutions across the United States. During the three-week survey period, two reminders were sent to the same email discussion lists and community discussion boards.

Analysis of the seventy-five viable surveys responses was carried out using Excel's Data Analysis tool.

Nineteen survey respondents agreed to participate in a follow-up interview, and seven accepted the subsequent interview invitation. The interview included eleven questions (supplemental Appendix B) and lasted thirty to forty-five minutes. Most interviews were held virtually with GoToMeeting software between February 18–25, 2020, although one interviewee answered the questions via email.

## RESULTS

The survey received eighty-one responses. Five respondents who answered “no” to the question “Are you a librarian responsible for nursing and/or allied health services or collections at your community college?” were removed from the analysis, as they did not meet the minimum requirement. One duplicate survey respondent was also removed from the analysis.

### Respondent demographics

The 75 survey respondents were community college librarians who were responsible for health sciences collections and services. Demographic information obtained by the survey included job title, position (full time versus part time), rank (faculty versus non-faculty), and all college degrees (Table 1). Most (65%) respondents did not have an advanced degree beyond the ALA-accredited master's of library or information science degree (MLS/MIS). When asked about the major of their advanced degrees beyond the MLS/MIS, 2 respondents had advanced degrees in the natural or health sciences, and 24 had an advanced degree in education, instructional design, or the humanities. Respondents had worked as a community college librarian for 6–10 years (24%), 1–5 years (23%), 11–15 years (19%), 16–20 years (16%), more than 22 years (11%), or less than 1 year (8%).

**Table 1:** Demographics of respondents

Demographics	Total	
	n	%
Job title		
Librarians	57	76%
Directors/deans	14	19%
Administrators	2	3%
Other	2	3%
Total	75	
Position		
Full time	71	95%
Part time	4	5%
Total	75	
Rank		
Faculty	43	57%
Nonfaculty	32	43%
Total	75	
College degrees		
Associate degree	7	9%
Bachelor of arts	35	47%
Bachelor of science	15	20%
Master of library or information science (MLS or MIS)	72	96%
Master of arts or science	12	16%
Master of science in nursing	0	—
Master of public health	0	—
Law degree (JD, LLM, SJD)	0	—
Doctorate of education (EdD)	1	1%
Doctorate of health sciences (DH Sc or DHS)	0	—
Doctorate in nursing practice (DNP)	0	—
Doctorate of philosophy (PhD)	0	—
Medical degree (MD, DO, DDS, DVM)	0	—
Other	12	16%

Most (96%) respondents worked at a public community college, 4% worked at a private community college, and no responses were received from tribal community college librarians. Nearly one-third (29%) of respondents reporting working in a library with 2 or 3 full-time librarians, 24% worked with 4–5 librarians, 23% worked as solo librarian, 19% worked with 8 or more librarians, and 5% worked with 6–7 librarians.

The top 7 health sciences programs offered by the respondents' community colleges were nursing (89%), health sciences (64%), emergency medical technician and/or paramedic (57%), radiologic technology or magnetic resonance imaging technology (49%), medical billing and coding (44%), medical assistance (43%), and physical therapy assistance (40%). Respondents served a minimum of 1 and a maximum of 21 health-related programs.

Follow-up interviews demonstrated that many respondents did not solely serve health sciences programs but had diverse liaison collection development roles with multiple non-health sciences–related areas such as business, computer science, criminal justice, English, environmental science, funeral science, horticulture, industrial technology, math, and science.

Slightly more than half (53%) of respondents had no knowledge of the MLA competencies, 36% knew about the MLA competencies, and 11% were knowledgeable about the MLA competencies and applied them to their work.

### MLA competencies

**Figure 1:**
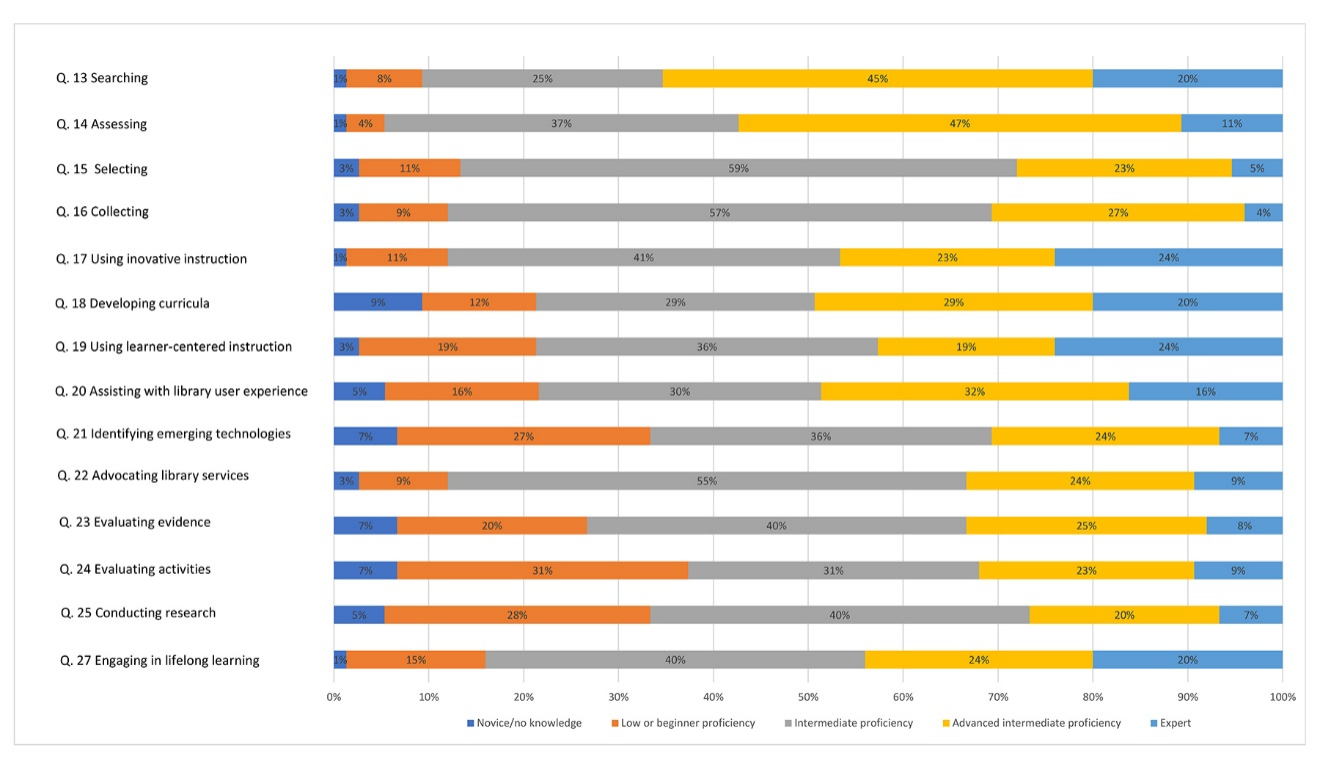
Self-reported MLA competencies and sub-competencies

The survey included 14 questions asking respondents to self-report their professional competencies and sub-competencies based on the MLA Competencies Self-Assessment quiz (Figure 1). Respondents had a median score of 4 (advanced intermediate proficiency) for questions 13, 14, and 18 and a median score of 3 (intermediate proficiency) for questions 15–17, 19–25, and 27. No questions had a median score of 5 (expert), 2 (low or beginning proficiency), or 1 (novice/no knowledge).

Competency 6 focuses on promoting the development of the health information professional and collaborating with other professionals to improve health care and access to health care information. Related to this competency, 2 questions asked about lifelong learning. Respondents selected networking with other librarians (65%) and attending state, regional, or national conferences (63%) as their top 2 choices for furthering their engagement as a health sciences librarian. Fewer respondents reported presenting at conferences (25%) or teaching credit courses (15%). Very few participants had completed MLA specializations (7%), and only 1% achieved MLA Academy of Health Information Professionals (AHIP) membership. When asked whether they used the MLA competencies to further their current position as a health sciences librarian and demonstrate the value of their library, 75% of respondents said “no.”

### Professional engagement

To further understand community college health sciences librarians' engagement in the profession, the survey included 2 questions specifically about engagement. When asked about their membership in professional associations with the option to select more than 1 answer, slightly more than half (53%) of respondents chose ALA. Nearly one-third (29%) stated that they were not a member of any health sciences association. Others reported being a member of a state health sciences association (16%), regional health sciences association (15%), or local health sciences association (13%). Only 7% of respondents were members of MLA. “Other” comments (12%) included membership in the National Network of Library of Medicine (NNLM).

When asked to report their regular attendance at health sciences conferences, 60% of respondents stated that they did not attend conferences, 23% attended local conferences, 19% attended state conferences, 13% attended national conferences, and 12% attended regional conferences.

In follow-up interviews, respondents described their involvement in the profession to keep abreast of new trends. They discussed reading email discussion lists, joining library organizations, attending webinars, and using library organization resources to become or stay involved in the profession. Webinars offered by the National Library of Medicine and NNLM regions were a common resource. Additionally, respondents mentioned collaborating with local networks, with one respondent stating they were “part of a consortium that includes community colleges and tribal libraries to help with negotiating database costs and discussions about topics.”

### Barriers to professional engagement

The last section of the survey addressed barriers, if any, to engaging with the professional community. Respondents answered three questions about barriers relating to attending local, state, and national conferences; continuing their education; and publishing, presenting, and teaching credit courses. Top barriers experienced by respondents included lack of financial support, small library staff, schedule conflicts, and lack of release time (Table 2).

**Table 2:** Barriers to professional engagement

	Q. 31 Conferences	Q. 32 Continuing education	Q. 33 Publishing/presenting
Financial	73%	41%	36%
Small staff	67%	41%	44%
Schedule conflict	43%	44%	37%
Release time	41%	25%	43%
No support from my administration	23%	8%	27%
I do not experience any barriers	9%	27%	20%
I do feel valued in the sponsoring association	9%	NA	NA
Other	8%	7%	1%
No interest	5%	1%	19%
Technology	1%	NA	NA
I lack skills in this area	NA	NA	16%

One theme that was evident in the open-ended final survey question was that community college librarians, in contrast to other types of academic librarians, felt more thinly spread across multiple unrelated disciplines. Unlike the common academic library trend to have subject specialists in just a few disciplines, community college librarians are often responsible for many disciplines across large dispersed campus systems. One respondent stated that “we have a student population in excess of 25,000 and yet approximately the same number of librarians that we had when enrollment was 8,000. We now have multiple campuses to support with five librarians who conduct instruction.” Another said, “besides being the health science librarian at my campus (we are 4 campuses), I am also the acquisitions librarian for all campus libraries.” Echoing this, another respondent stated, “health sciences and allied health is important, but only one area I need to select for.”

## DISCUSSION

This study provided crucial insights into community college health sciences librarians' perceived professional competencies. The results affirmed that community college librarians who were responsible for collections and services in the health sciences met the MLA competencies, with most respondents reporting intermediate or advanced intermediate proficiency in specific competencies and sub-competencies. However, these findings implied the potential for continued improvement in professional skills and abilities to raise their competency levels to advanced proficiency or higher.

MLA places great emphasis on the importance of competencies by promoting competency self-assessment and offering numerous professional development courses based on specific competencies [5]. Several studies supported the development of health sciences librarian competencies by on-the-job training, continuing education courses, and review of their MLS curricula [6–8]. Another study reiterated the importance of continued development of skills and abilities of health sciences librarians based on the changing landscape of their users [13].

The survey included questions about respondents' engagement with the profession to continue building their knowledge and develop competencies in the health sciences. The results showed that community college librarians who were responsible for health sciences collections and services were very engaged in the profession: most were members of ALA or other non-health-related library associations. Their development, engagement, and commitment to the profession occurred when they continued their education and talked to peers as well as nursing and allied health faculty, who were all part of their communities. Many respondents added that they attended NNLM webinars for additional training and engagement. During follow-up interviews, community college librarians described attending conferences, taking continuing education courses, and networking with both librarians and faculty members as modes of advancing their competencies while on the job. Therefore, to cultivate community college health sciences librarians' continued progress in MLA professional competencies, employers can provide release time, financial support, and encouragement.

The findings of the present study supported those of several previous studies concerning barriers that librarians faced in meeting professional competencies. Barriers that were identified in the present study included lack of funding; low staffing; schedule conflicts when attending conferences, continuing education webinars, and courses; and challenges involved in publishing and presenting at conferences. These same barriers were identified in a study that focused on rural and small libraries, including budget cuts as a factor [10]. Another study focused on delivery preference of professional development classes and how to overcome barriers related to cost by promoting online synchronous and asynchronous classes [9]. Barriers to professional development related to training and institutional support [11] can be overcome by building a culture of support and increasing the availability of low-cost continuing education sessions [14]. Adding to this list of barriers, community college librarians have many different responsibilities and, in some cases, are dispersed across many campuses, with an insufficient number of full-time librarians to share job responsibilities.

This study advocates several recommendations for community college health sciences librarians to achieve advanced or higher proficiencies. Numerous opportunities exist to advance skills and abilities in all areas with local partners. MLA offers a combination of self-paced courses and webinars for a fee [15]. Many MLA chapters and state health sciences associations receive funding to offer members free access to these webinars. NNLM offers numerous free self-paced courses and webinars in all areas of health sciences, including specialization certificates [16, 17].

Community college librarians can also continue to develop their skills in evidence-based research and practice to apply these skills to decision making, to identify and develop evaluation methods and metrics to improve services, and to publish. Now more than ever, libraries need to substantiate their value to their institutions with evidence [18]. Evidence-based research and practice can be accomplished by learning to assess their programs, activities, services, and collections in the health sciences to advocate for the value of their libraries. Barriers that community college librarians encounter can be overcome with free or low-cost webinars and continuing education opportunities, virtual options for attending conferences, and educational support or mentors to support publishing and presenting at conferences.

A limitation of this study is the survey response rate. The actual size of the community college health sciences librarian population in the United States is unknown, so it is unclear to what extent the present results can be generalized. Also, a low survey response rate does not necessarily indicate low interest in the topic [19]. Furthermore, respondents self-assessed their levels of competencies and could have understated or overstated them.

This study contributes to understanding how health sciences community college librarians develop professional competencies, as defined by the *MLA Competencies for Lifelong Learning and Professional Success,* and specific barriers that impede their development despite their professional engagement. These findings can help MLA and other organizations such as NNLM and ALA improve their educational opportunities, outreach, and value to community college librarians, a previously overlooked population of health sciences librarians.

## Data Availability

Data associated with this article are available in the Open Science Framework at https://osf.io/vq4zy/?view_only=c2e1793a0ce249cdb0e52c84f8872af1.

## SUPPLEMENTAL FILES

**Appendix A:** Survey questionnaire

**Appendix B:** Follow-up interview questions
