# Supplementary material for: Health sciences librarian competency perceptions: a survey of national community college librarians
Source: J Med Libr Assoc. 2021 Jan 1;109(1):44–51. doi: 10.5195/jmla.2021.994 (PMC7772980; doi:10.5195/jmla.2021.994)
Supplement: Supplementary file 1 — Appendix A: Survey questionnaire [file jmla-109-1-44-s01.pdf]

JMLA Journal of the Medical Library Association

SC
Supplemental content to
J Med Libr Assoc. Jan;109(1):dx.doi.org/10.5195/jmla.2021.994
www.jmla.mlanet.org
© McCarthy 2021

Health sciences librarian competency perceptions: a survey of national community college librarians

Sandra C. McCarthy

APPENDIX A

Survey questionnaire

Informed consent

I am researching community college health sciences librarians' perceptions of Medical Library Association (MLA) competency skills.

Purpose: The purpose of this study is to determine if community college health sciences librarians self-report having the essential skills, knowledge, and abilities that are necessary for health information professionals' practice as determined by the *MLA Competencies for Lifelong Learning and Professional Success.* In addition, the study will identify what barriers, if any, community college health sciences librarians have and how engaged community college health sciences librarians are with developing their competencies.

Study procedures:

1. Participants will complete this survey to ascertain how they think their existing skills, knowledge, and abilities compare to those listed in the *MLA Competencies for Lifelong Learning and Professional Success.* The survey will also include some demographic questions and ask about any barriers participants face in meeting these competencies and engaging in the health sciences libraries profession. This survey will take approximately fifteen to twenty minutes to complete.
2. The survey is anonymous, and participants will be asked at the end to leave an email address if they wish to volunteer for a follow up interviews, approximately three weeks to five weeks after they complete the survey. The interview will take thirty minutes to one hour, either face-to-face or virtually using GoToMeeting. Data from independent interviews will be thematically coded and reported out as an aggregate chart.

Institutional board approval: This study has received institutional review board (IRB) approval.

Benefits: The results of the study will be shared in a future journal article or presented at a conference. Benefits to your participation in the survey may include: learning more about the MLA health sciences librarians' competencies framework, identifying areas of improvement for your professional practice as a health sciences librarian, and reporting barriers to improving health sciences librarian competencies and engaging with the broader medical library community that are specific to the community college setting. The benefits for society are that the study will fill a gap in the literature about how health sciences community college librarians develop librarianship competencies as defined by the *MLA Competencies for Lifelong Learning and Professional Success* and will identify any barriers encountered that impede their development. Learning this may help MLA improve its educational offerings, outreach, and value to community college librarians, a traditionally understudied community of health sciences librarians.

Risks: There are no known risks currently to participate in this research study.

JMLA Journal of the Medical Library Association

SC
Supplemental content to
J Med Libr Assoc. Jan;109(1):dx.doi.org/10.5195/jmla.2021.994
www.jmla.mlanet.org
© McCarthy 2021

Costs: There are no costs to you for participation in this research study.

Compensation: There is no compensation for completing the survey.

Confidentiality: No identifiable information will be collected. Responses will be completely anonymous. The researcher will not collect any personal- or institutional-identifying data, nor will the researcher analyze or report data in any way that might compromise individual identity.

If you volunteer to be part of the follow-up interviews, you will be asked to provide your name, email, and phone number. Contact information will only be used for a follow-up interview. Your contact information will be removed from survey analysis and deleted after the interview is complete. The interview data will not identify you nor your community college in the results. Anonymized interview data will be stored on a hard drive that is in a locked room that is accessible only to the researcher. Data will be retained for two years.

Voluntary participation/withdrawal: Taking part in this research study is voluntary. You may choose not to participate, or if you decide to participate, you can change your mind later and withdraw from the study.

Questions: If you have any questions about this study now or in the future, you may contact the lead researcher by phone or by email.

Please complete the survey by January 31, 2020.

Participation: By completing this survey, you are agreeing to participate in this research study. Additionally, participation in this research is for community college health sciences librarians of the United States over the age of eighteen; if you are not a community college health sciences librarian of the United States and/or are under the age of eighteen, please do not complete this survey.

I have read and understand this informed consent and agree to continue.

○ Yes
○ No

Survey questionnaire

A community college health sciences librarian is defined as an information professional, librarian, or informaticist who has special knowledge in quality health information resources and is responsible for at least one health sciences program.

1. Are you a librarian responsible for nursing and/or allied health services or collections at your community college?

○ Yes
○ No [a no answer means the survey ends for this librarian]

JMLA Journal of the Medical Library Association

SC
Supplemental content to
J Med Libr Assoc. Jan;109(1):dx.doi.org/10.5195/jmla.2021.994
www.jmla.mlanet.org
© McCarthy 2021

2. What is your role at your institution?
- Librarian
- Director and/or dean
- Administrator (department coordinator or manager)
- Other:

3. Is your position:
- Full time
- Part time

4. Do you have faculty rank/status?
- Yes
- No

5. What degrees do you hold? [check all that apply]
- Associate degree
- Bachelor of arts
- Bachelor of science
- Master of library or information science
- Master of arts or science
- Master of science in nursing
- Master of public health
- Law degree (JD, LLM, SJD)
- Doctorate of education (EdD)
- Doctorate of health sciences (DH Sc or DHS)
- Doctorate in nursing practice (DNP)
- Doctorate of philosophy (PhD)
- Medical degree (MD, DO, DDS, DVM)
- Other:

6. What is your undergraduate degree subject major? [open text box]

7. If you have an advanced degree(s) other than a master of library or information science, what is your degree subject major(s)? [open text box]

8. What health programs are you responsible for providing services and collections to? [check all that apply]
- Cardiovascular technicians/technologists
- Clinical lab technicians/technologists
- Dental assistants
- Dental hygienists

JMLA Journal of the Medical Library Association
Supplemental content to
J Med Libr Assoc. Jan;109(1):dx.doi.org/10.5195/jmla.2021.994
www.jmla.mlanet.org
© McCarthy 2021

- Emergency medical technicians (EMTs) and paramedics
- Exercise physiologists
- Health sciences professionals
- Home health aides and personal care aides
- Massage therapists
- Medical assistants
- Medical billing and coding professionals
- Medical transcriptionists
- Nuclear medicine technologists
- Nursing professionals
- Occupational health and safety specialists and technicians
- Occupational therapy assistants
- Opticians
- Pharmacy technicians
- Phlebotomists
- Physical therapist assistants
- Radiation therapists
- Radiologic technologists/MRI technologists
- Respiratory therapists
- Sonographer technicians/technologists
- Surgical technologists
- Veterinary technologists and technicians
- Other:

9. How many full-time librarians work at your community college library?

- Solo librarian
- 2–3
- 4–5
- 6–7
- 8 or more

10. How long have you been a community college librarian?

- Less than 1 year
- 1 year–5 years
- 6 years–10 years
- 11 years–15 years
- 16 years–20 years
- More than 21 years

JMLA Journal of the Medical Library Association

SC

Supplemental content to
J Med Libr Assoc. Jan;109(1):dx.doi.org/10.5195/jmla.2021.994
www.jmla.mlanet.org
© McCarthy 2021

11. Your community college is considered a:
- ○ Public
- ○ Independent
- ○ Tribal

12. Which statement best describes your knowledge about MLA's *Competencies for Lifelong Learning and Professional Success?*
- ○ I have no knowledge about the MLA competencies
- ○ I know about the MLA competencies
- ○ I am knowledgeable about the MLA competencies and apply them to my role as a health sciences librarian

Competency 1: Information Services

A health information professional locates, evaluates, synthesizes, and delivers authoritative information in response to biomedical and health inquiries.

13. Which statement best describes your skills in searching health sciences databases and other online resources?
- ○ Novice/no knowledge
- ○ Low or beginner proficiency: I can describe steps in conducting a search; I can identify relevant resources; I can formulate search strategies using appropriate search construction techniques, subject descriptors, and natural language and Boolean connectors.
- ○ Intermediate proficiency: I can apply steps in conducting a search; I can identify relevant resources; I can formulate search strategies using appropriate search construction techniques, subject descriptors, and natural language and Boolean connectors.
- ○ Advanced intermediate proficiency: I am skillful with formulating and executing complex search strategies in a variety of information resources; I can customize search outputs.
- ○ Expert: I can formulate and execute complex search strategies in a variety of information resources; I can customize search outputs.

14. Which statement best describes your skills in assessing information needs for health sciences?
- ○ Novice/no knowledge
- ○ Low or beginner proficiency: I can use reference interview skills.
- ○ Intermediate proficiency: I can apply reference interviews.
- ○ Advanced intermediate proficiency: I am skillful in conducting reference interviews; I am skillful with the language of biomedical science.
- ○ Expert: I can use the language of biomedical science in conducting reference interviews.

15. Which statement best describes your skills in selecting information for health sciences?
- ○ Novice/no knowledge
- ○ Low or beginner proficiency: I can locate published information and assess its authority, accuracy, objectivity, currency, and relevance.

SC
Supplemental content to
J Med Libr Assoc. Jan;109(1):dx.doi.org/10.5195/jmla.2021.994
www.jmla.mlanet.org
© McCarthy 2021

- Intermediate proficiency: I am qualified in locating published information and assess its authority, accuracy, objectivity, currency, and relevance.
- Advanced intermediate proficiency: I am skillful with unpublished resources and consult with subject experts.
- Expert: I can use unpublished resources and consults with subject experts.

Competency 2: Information Management

A health information professional curates and makes accessible bioscience, clinical, and health information data, information, and knowledge.

16. Which statement best describes your skills in selecting, acquiring, managing, evaluating, and circulating bioscience, clinical, and health information?

- Novice/no knowledge
- Low or beginner proficiency: I can describe collection management tools.
- Intermediate proficiency: I can apply collection management tools.
- Advanced intermediate proficiency: I am skillful with current evaluation methodologies, including bibliometrics, to develop and implement collection management policies and strategies.
- Expert: I can use current evaluation methodologies, including bibliometrics, to develop and implement collection management policies and strategies.

Competency 3: Instruction & Instructional Design

A health information professional educates others in the skills of bioscience, clinical, and health information literacy.

17. Which statement best describes your skills with using innovative instructional and communication methods and technologies?

- Novice/no knowledge
- Low or beginner proficiency: I can describe trends in communication and instructional methods and technologies; I can use social media and web-based technologies.
- Intermediate proficiency: I can apply trends in communication and instructional methods and technologies; I can use social media and web-based technologies.
- Advanced intermediate proficiency: I am skillful with evaluating, developing, and implementing innovative instructional and communicating strategies and technologies.
- Expert: I can evaluate, develop, and implement innovative instructional and communication strategies and technologies.

18. Which statement best describes your skills in developing curricula using contemporary instructional design principles?

- Novice/no knowledge
- Low or beginner proficiency: I can describe principles of instructional design.
- Intermediate proficiency: I can apply principles of instructional design.

© McCarthy 2021

- Advanced intermediate proficiency: I am skillful with developing face-to-face and/or online learning activities based on instructional design principles.
- Expert: I can develop face-to-face and online learning activities based on instructional design principles.

19. Which statement best describes your skills in using learner-centered instructional approaches?
- Novice/no knowledge
- Low or beginner proficiency: I can describe learner-centered instructional approaches.
- Intermediate proficiency: I can apply learner-centered instructional approaches.
- Advanced intermediate proficiency: I am skillful with learner-centered face-to-face and online learning approaches.
- Expert: I can use learner-centered face-to-face and online learning approaches.

Competency 4: Leadership & Management

A health information professional manages personnel, time, budget, facilities, and technology and leads others to define and meet institutional goals.

20. Which statement best describes your skills in developing and implementing enhancements to the library user experience as it relates to health sciences collections and services?
- Novice/no knowledge
- Low or beginner proficiency: I can describe the literature on library user experience and user experience assessment.
- Intermediate proficiency: I can apply literature on library user experience and user experience assessment.
- Advanced intermediate proficiency: I am skillful with using results of formal and informal user experience assessments to propose and implement library user experience enhancements.
- Expert: I can use results of formal and informal user experience assessments to propose and implement library user experience enhancements.

21. Which statement best describes your skills in identifying emerging technologies in health sciences and advocating for their use?
- Novice/no knowledge
- Low or beginner proficiency: I can explore and evaluate emerging technologies.
- Intermediate proficiency: I can apply and use emerging technologies.
- Advanced intermediate proficiency: I am skillful with leading initiatives to incorporate new technologies.
- Expert: I can lead initiatives to incorporate new technologies.

22. Which statement best describes your skills in developing and implementing effective advocacy, marketing, and communication strategies for the health sciences collection?
- Novice/no knowledge
- Low or beginner proficiency: I can promote institutional mission and goals; I can form internal partnerships.

JMLA Journal of the Medical Library Association

SC
Supplemental content to
J Med Libr Assoc. Jan;109(1):dx.doi.org/10.5195/jmla.2021.994
www.jmla.mlanet.org
© McCarthy 2021

○ Intermediate proficiency: I can apply institutional mission and goals to health sciences; I use internal partnerships.
○ Advanced intermediate proficiency: I am skillful with designing marketing and public relations strategies and programs; I can form external partnerships.
○ Expert: I can design marketing and public relations strategies and programs; I can form external partnerships.

Competency 5: Evidence-Based Practice & Research

A health information professional evaluates research studies, uses research to improve practice, conducts research, and communicates research results.

23. Which statement best describes your skills in finding and evaluating evidence to support decision making in the health sciences?
○ Novice/no knowledge
○ Low or beginner proficiency: I can describe evidence-based practice; I can formulate questions; I can develop search strategies; I can locate relevant, credible, and transferable published evidence.
○ Intermediate proficiency: I can apply evidence-based practice; I can use research questions; I can apply search strategies; I can use relevant, credible, and transferable published evidence.
○ Advanced intermediate proficiency: I am skillful with using evidence to make and justify decisions.
○ Expert: I can use evidence to make and justify decisions.

24. Which statement best describes your skills in evaluating activities, programs, collections, and services using evidence-based methodologies?
○ Novice/no knowledge
○ Low or beginner proficiency: I can gather data and user input on activities and services.
○ Intermediate proficiency: I can apply data and user input on activities and services.
○ Advanced intermediate proficiency: I am skillful with identifying and developing evaluation methods and metrics for assessing and improving services.
○ Expert: I can identify and develop evaluation methods and metrics for assessing and improving services.

25. Which statement best describes your skills in conducting research?
○ Novice/no knowledge
○ Low or beginner proficiency: I can describe the research process, structure of research papers, and common research methods, including bibliometrics; I can explain standards of ethical research.
○ Intermediate proficiency: I can apply and use the research process, structure of research papers, and common research methods, including bibliometrics; I can apply standards of ethical research.
○ Advanced intermediate proficiency: I am skillful with selecting and implementing appropriate research design; collecting, managing, and analyzing data; interpreting results; and explaining threats to validity of conclusions.
○ Expert: I can select and implement appropriate research design; collect, manage, and analyze data; interpret results; explain threats to validity of conclusions.

SC
Supplemental content to
J Med Libr Assoc. Jan;109(1):dx.doi.org/10.5195/jmla.2021.994
www.jmla.mlanet.org
© McCarthy 2021

Competency 6: Health Information Professionalism

A health information professional promotes the development of the health information professions and collaborates with other professionals to improve health care and access to health care information.

26. To further engage in health sciences library profession, do you? [check all that apply]

- Publish articles
- Attend conferences (state, regional, or national)
- Present at conferences (poster, lighting round, round table, presentation)
- Network with other health sciences librarians and/or community college librarians
- Teach credit classes
- Contribute to research
- Serve on health sciences library association committees
- Obtain MLA Academy of Health Information Professionals (AHIP) membership
- Obtain MLA Consumer Health Information Specialization (CHIS)
- Obtain MLA Disaster Information Specialization
- I do not engage
- Other: [text box]

27. Which statement best describes your skills in participating in and fostering a culture of lifelong learning?

- Novice/no knowledge
- Low or beginner proficiency: I can identify gaps in knowledge and skills; I can seek professional development opportunities and mentor to address gaps.
- Intermediate proficiency: I can apply gaps in knowledge and skills; I use professional development opportunities and mentor to address gaps.
- Advanced intermediate proficiency: I am skillful with building professional skills in advance of emerging trends.
- Expert: I can build professional skills in advance of emerging trends; I can mentor others.

The following questions relate to engagement in the profession and what barriers, if any, you encounter.

28. To further engage with health sciences profession, are you a member of? [check all that apply]

- My local health sciences association
- My state health sciences association
- My regional health sciences association
- Medical Library Association
- American Library Association
- I am not a member of any health sciences association
- Other:

29. To further engage as a health sciences librarian, do you regularly attend health sciences conferences at the following levels? [check all that apply]
- O Local
- O State
- O Regional
- O National
- O I do not attend conferences

30. Do you use the MLA professional competencies framework to further your current position as a health sciences librarian and demonstrate the value of your library?
- O Yes
- O No

31. What, if any, barriers do you face attending conferences (local, state, regional, national)? [check all that apply]
- O Financial
- O Time release
- O Schedule conflict
- O Small staff
- O Technology
- O No support from my administration
- O No interest
- O I do not feel valued in the sponsoring association
- O I do not experience any barriers
- O Other:

32. What, if any, barriers do you face in participating in continuing education webinars/courses? [check all that apply]
- O Financial
- O Release time
- O Schedule conflict
- O Small staff
- O No support from administration
- O No interest
- O I do not experience any barriers
- O Other:

33. What, if any, barriers do you face with publishing, teaching credit courses, presenting at conferences (paper, poster, or presentation), and/or serving on committees? [check all that apply]
- O Financial
- O Time release

JMLA Journal of the Medical Library Association

SC
Supplemental content to
J Med Libr Assoc. Jan;109(1):dx.doi.org/10.5195/jmla.2021.994
www.jmla.mlanet.org
© McCarthy 2021

- Schedule conflict
- Small staff
- No support from administration
- I lack the skills in this area
- No interest
- I do not experience any barriers
- Other:

34. Use the textbox to share any additional comments: [open text box]

Are you interested in participating in a follow up interview? If you are interested in and available to speak with the researcher further about this subject, please provide your name, phone number, and email address.
Name:
Phone number:
Email:

Thank you for participating in this survey.
