# Supplementary material for: Health sciences librarian competency perceptions: a survey of national community college librarians
Source: J Med Libr Assoc. 2021 Jan 1;109(1):44–51. doi: 10.5195/jmla.2021.994 (PMC7772980; doi:10.5195/jmla.2021.994)
Supplement: Supplementary file 2 — Appendix B: Follow-up interview questions [file jmla-109-1-44-s02.pdf]

JMLA Journal of the Medical Library Association

SC
Supplemental content to
J Med Libr Assoc. Jan;109(1):dx.doi.org/10.5195/jmla.2021.994
www.jmla.mlanet.org
© McCarthy 2021

Health sciences librarian competency perceptions: a survey of national community college librarians

Sandra C. McCarthy

APPENDIX B

Follow-up interview questions

- How proficient are community college health sciences librarians in the Medical Library Association (MLA) competencies self-assessment?
- What barriers do community college health sciences librarians face with developing their competencies?
- How engaged are community college health sciences librarians in continuing education?

Interview questions
Date:
Interviewee:

1. Describe your path to becoming a community college health sciences librarian?

2. List your collection development liaison areas [include both health sciences and other subject areas]:

3. Describe how you stay abreast with new trends in health sciences librarianship?

4. Referring to the MLA *Competencies for Lifelong Learning and Professional Success:* As a health provider, do you use the performance criteria for each competency to assess whether your institution is delivering service at the highest possible level?

5. As a health sciences librarian, are you part of the health sciences program accreditation process at your community college?

6. As a health sciences librarian, what services do you provide your faculty and/or students?

7. Do you value networking with other community college librarians or health sciences?

8. What barriers, if any, do you encounter with being engaged in the health sciences librarianship profession? This can be with having an association membership; attending conferences; publishing; presenting either a poster, talk, or lighting round; other…

9. If you experience any barriers, how do you overcome your barriers?

JMLA Journal of the Medical Library Association

Supplemental content to
J Med Libr Assoc. Jan;109(1):dx.doi.org/10.5195/jmla.2021.994
www.jmla.mlanet.org
© McCarthy 2021

10. How can the library associations (MLA, the American Library Association, or state associations) increase involvement by community college librarians?

11. What do you enjoy most about being a health sciences librarian?
